# The role of radiation‐induced charge imbalance on the dose‐response of a commercial synthetic diamond detector in small field dosimetry

**DOI:** 10.1002/mp.13542

**Published:** 2019-05-02

**Authors:** Hui Khee Looe, Daniela Poppinga, Rafael Kranzer, Isabel Büsing, Tuba Tekin, Ann‐Britt Ulrichs, Björn Delfs, Dennis Vogt, Jan Würfel, Björn Poppe

**Affiliations:** ^1^ University Clinic for Medical Radiation Physics Medical Campus Pius Hospital Carl von Ossietzky University Oldenburg Germany; ^2^ PTW Freiburg Freiburg Germany

**Keywords:** diamond detector, polarity effect, radiation‐induced charge imbalance, small fields, volume effect

## Abstract

**Purpose:**

Discrepancy between experimental and Monte Carlo simulated dose–response of the microDiamond (mD) detector (type 60019, PTW Freiburg, Germany) at small field sizes has been reported. In this work, the radiation‐induced charge imbalance in the structural components of the detector has been investigated as the possible cause of this discrepancy.

**Materials and methods:**

Output ratio (OR) measurements have been performed using standard and modified versions of the mD detector at nominal field sizes from 6 mm × 6 mm to 40 mm × 40 mm. In the first modified mD detector (mD_reversed), the type of charge carriers collected is reversed by connecting the opposite contact to the electrometer. In the second modified mD detector (mD_shortened), the detector's contacts have been shortened. The third modified mD detector (mD_noChip) is the same as the standard version but the diamond chip with the sensitive volume has been removed. Output correction factors were calculated from the measured OR and simulated using the EGSnrc package. An adapted Monte Carlo user‐code has been used to study the underlying mechanisms of the field size‐dependent charge imbalance and to identify the detector's structural components contributing to this effect.

**Results:**

At the smallest field size investigated, the OR measured using the standard mD detector is >3% higher than the OR obtained using the modified mD detector with reversed contact (mD_reversed). Combining the results obtained with the different versions of the detector, the OR have been corrected for the effect of radiation imbalance. The OR obtained using the modified mD detector with shortened contacts (mD_shortened) agree with the corrected OR, all showing an over‐response of less than 2% at the field sizes investigated. The discrepancy between the experimental and simulated output correction factors has been eliminated after accounting for the effect of charge imbalance.

**Discussions and conclusions:**

The role of radiation‐induced charge imbalance on the dose–response of mD detector in small field dosimetry has been studied and quantified. It has been demonstrated that the effect is significant at small field sizes. Multiple methods were used to quantify the effect of charge imbalance with good agreement between Monte Carlo simulations and experimental results obtained with modified detectors. When this correction is applied to the Monte Carlo data, the discrepancy from experimental data is eliminated. Based on the detailed component analysis using an adapted Monte Carlo user‐code, it has been demonstrated that the effect of charge imbalance can be minimized by modifying the design of the detector's contacts.

## 1 Introduction

Since the commercialization of the microDiamond (mD) detector (type 60019, PTW Freiburg, Germany), the dose‐response of the detector in small fields has been investigated extensively using experimental methods,^1^, ^2^, ^3^, ^4^ Monte Carlo simulations,^5^, ^6^, ^7^ or a combination of both.^8^, ^9^, ^10^ Although these studies have demonstrated that the detector is suitable for small field dosimetry, deviations of the output ratios (OR) from the field output factors at small field sizes have been reported. The cause can be mainly attributed to two perturbation effects: (a) the volume‐averaging due to the finite area of the sensitive volume (2.2 mm diameter); and (b) the density perturbation due to the higher electron density of the detector's structural components compared to that of normal water.^11^, ^12^, ^13^, ^14^, ^15^, ^16^, ^17^, ^18^

The perturbation due to the volume‐averaging effect is well‐understood since the measured signal represents the weighted average of the absorbed dose to water over the sensitive volume of the detector. The volume‐averaging correction factor, *P*_vol_, can be derived as the ratio of the absorbed dose to water at the point of measurement and this average dose value.^13^, ^19^, ^20^, ^21^ At small field sizes, the volume‐averaging effect will cause the detector to under‐respond along the central axis due to the bell‐shaped dose profiles, that is, *P*_vol_ is larger than unity.

On the other hand, the different physical mechanisms underlying the density perturbation effect have been elucidated by many authors. One of the possible disturbance mechanisms is the under‐ or overproduction of secondary electrons within the radiation‐sensitive material of the detector (the “insider effect”), compared to water of normal density.^11^, ^12^, ^13^, ^17^ Looe et al.^12^ also showed that the density of the structural materials, adjacent to the sensitive layer of a photon beam dosimetry detector on the upstream or downstream side, can cause perturbation of the fluence profiles of the secondary electrons hitting this sensitive layer. In fact, at large field sizes, it has been shown that the secondary electron fluence in the detector volume is independent of the density of its surrounding components indicating the achievement of secondary electron equilibrium according to Fano's theorem.^12^ However, with decreasing field size, the secondary electron fluence will be disturbed due to the missing inward transport of secondary electrons across the field boundary. Therefore, at small fields, where the condition of lateral secondary electrons’ equilibrium is not fulfilled, structural components with density higher than normal water surrounding the sensitive volume such as in the case of the mD detector will cause the detector to over‐respond, that is, the density perturbation correction factor is less than unity.

In fact, measurements using the mD detector have generally shown that the detector over‐responds at small field sizes.^1^, ^2^, ^3^, ^4^ In other words, the density effect would overweigh the volume‐averaging effect resulting in small field output correction factors less than unity. At field sizes of 4–5 mm dosimetric side length, the correction amounts to around 4–5%.^19^ Nevertheless, Monte Carlo studies often resulted in lower over‐response of the detector than observed in experimental studies. Many authors have therefore attempted to clarify the observed discrepancy between experimental and Monte Carlo studies. Andreo et al.^5^ have suggested that the sensitive area of the detector is smaller than the manufacturer's specification. They have shown that by reducing the sensitive area in the simulations and hence the associated volume‐averaging effect, better agreement between Monte Carlo and experimental results can be achieved. This proposition has since been disproved by three working groups.^22^, ^23^, ^24^ All studies used miniature beams to measure the sensitive area of the mD detector and have demonstrated that the sensitive area of the mD detector coincides with the manufacturer's specification within the experimental uncertainty. Recently, De Coste et al.^18^ have demonstrated that the output factors measured using the mD detector and a silicon diode are in good agreement (<1.6%) after applying Monte Carlo calculated small field correction factors for both detectors. Nevertheless, the discrepancy between the experimental and Monte Carlo simulated dose–response of the mD detector at small field sizes in the literature still requires further clarification. Indeed, the recommended correction factors in the TRS 483^19^ for the mD detector have been questioned recently due to the disagreements between the results reported in the literature so far.^25^, ^26^

In this work, we postulate that this discrepancy is partly caused by radiation‐induced charge imbalance in the metal contacts and cable of the mD detector. This aspect has not been studied so far as a possible contributor to the observed over‐response of mD detector at small field sizes. In a recent study,^27^ radiation‐induced charge imbalance in the inner electrode and cable has been identified as the main contributor to the observed field size‐dependent polarity effect of compact air‐filled ionization chambers. At small field sizes, the polarity effect of these microchambers is shown to increase with decreasing field size when they are oriented axially, that is, with their symmetrical axes oriented parallel to the beam's axis. If not properly accounted for, this effect is shown to cause discrepancies in the derivations of the small field output correction factors. A Monte Carlo user‐code has been developed to study the field size‐dependent polarity effect in terms of the radiation‐induced charge imbalance in different detector's components. Since the influence of charge imbalance on detector's signal has been shown previously to become more dominant with decreasing sensitive volume, it is noteworthy that the average dose–response of the mD detector is only 2.5 times higher than that of a PinPoint 3D chamber (type 31022, PTW Freiburg).

In this work, the role of radiation‐induced charge imbalance on the mD detector's dose–response at small field sizes has been investigated using multiple methods. Firstly, OR measurements using standard and modified versions of the mD detector were carried out. The output correction factors derived from the measured OR were then compared to Monte Carlo correction factors obtained by modeling the detector according to manufacturer's blueprint. Consequently, using an adapted Monte Carlo user‐code, the radiation‐induced charge imbalance in the detector's components and its role on the detector's dose–response were computed. This effect was compared to other perturbation effects such as the density perturbation effect and the volume‐averaging effect in terms of the associated correction factors.

## 2 Materials and methods

### 2.A. Radiation‐induced charge imbalance in the detector's components

Radiation detectors based on the principal of collection of free charge carriers released by ionizing radiation within the sensitive volume are commonly used in dosimetry. The most common of these are ionization chambers with two electrodes, where an external potential is applied between them to produce an electric field within the sensitive volume. One of these electrodes is acting as the collecting electrode, where the released free charge carriers of one sign will be collected and read out by an electrometer. Semiconductor detectors, such as a p‐n diode, or, in the case of the investigated mD detector, a Schottky diode, function similarly with the difference that the intrinsic electrical field is used to collect the charges and no external potential must be applied to the electrodes. The amount of charge collected is then used as a measure of the absorbed dose in the sensitive volume. Neglecting all possible effects influencing the charge collection, such as, ion recombination, temperature or polarity, the amount of charge collected, is proportional to the absorbed dose.

Recently, the radiation‐induced charge imbalance within conducting components that are connected between the detector's sensitive volume and the electrometer, for example, collecting electrode, contacts or cable, has been shown to contribute significantly to the so‐called polarity effect.^27^ When these components are being exposed to ionizing radiation, electrons released in these components can escape from them. This will cause an excess of holes (positive charges) within these detector's components, which are quantified by *Q* ^out^ computed as the number of excess holes times the elementary charge (1.6 × 10^−19^ C). On the other hand, electrons released in the surrounding medium can be stopped in these components causing an excess of negative charges, which are quantified by *Q* ^in^ computed as the number of excess electrons times the elementary charge of an electron (−1.6 × 10^−19^ C). The radiation‐induced net charge is then calculated as

(1) $$Q^{\mathrm{net}}=Q^{\mathrm{out}}+Q^{\mathrm{in}}$$

For example, *Q* ^net^ is negative, when there are excess negative charges in these detector components and vice versa.

The signal of a detector should represent solely the amount of charge released due to energy deposition (EDEP) of the ionizing radiation within the sensitive volume of the detector. This absolute amount of charge is denoted as |*Q* ^EDEP^|, which corresponds to the EDEP divided by the average energy required to produce one ion pair in the material, *w*, and multiplied by the elementary charge. The value of *w* is material dependent: 33.97 eV for air and 13.2 eV for diamond.^28^

The absolute amount of charge collected by the electrometer using positive and negative polarity are denoted as |*Q* ^+^| and |*Q* ^−^|, respectively. Taking the voltage‐independent polarity effect due to charge imbalance into account, these measured signals |*Q* ^+^| and |*Q* ^−^| are given by

(2) $$\left|Q^{+}\right|=\left|Q^{\mathrm{EDEP}}\right|+Q^{\mathrm{net}}$$

(3) $$\left|Q^{-}\right|=\left|Q^{\mathrm{EDEP}}\right|-Q^{\mathrm{net}}$$

The value of |*Q* ^EDEP^| due to EDEP in the detector's sensitive volume is obtained by combining Eqs. (2) and (3), which is the mean of the absolute signals obtained using positive and negative polarity.

(4) $$\left|Q^{\mathrm{EDEP}}\right|=\left(\left|Q^{+}\right|+\left|Q^{-}\right|\right)/2$$

**Figure 1:**
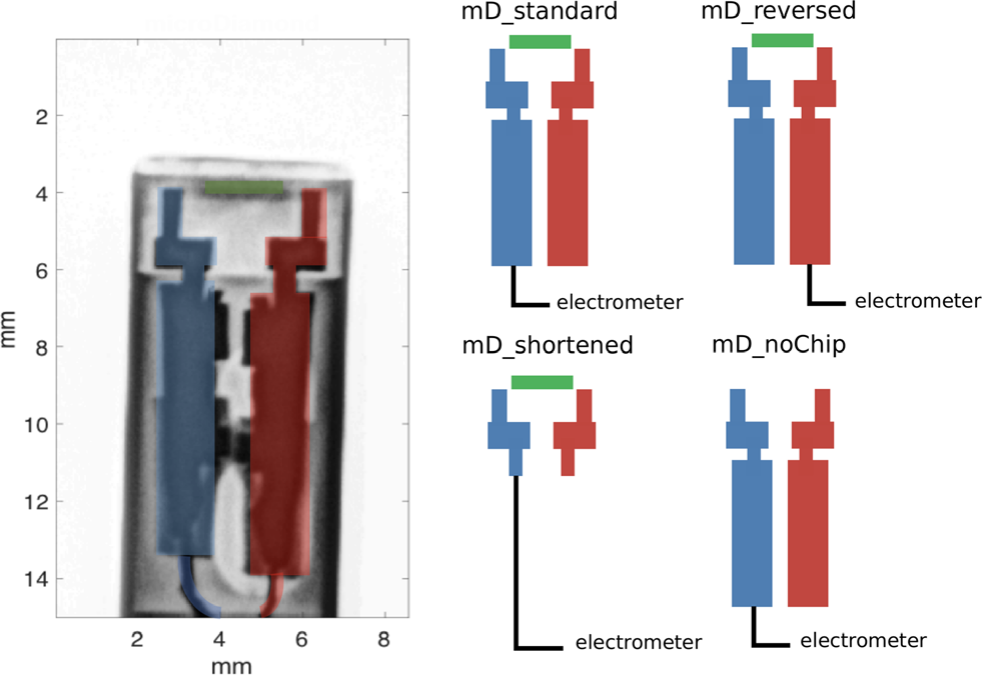
Left insert: x‐ray image of a standard microDiamond (mD) detector. The diamond chip with the sensitive volume is shown as a green line. The contacts of the detector are marked in blue and red. The type of charge carries collected depends on which contact is connected to the electrometer. Right insert: schematic drawings of the four versions of mD detector used in this work. [Color figure can be viewed at http://www.wileyonlinelibrary.com]

The same correction can be applied to the mD detector by considering that its polarity, that is, the type of charge carriers being collected, can be changed by reversing the contact connected to the electrometer. Figure 1 (left insert) shows an x‐ray image of the mD detector (adopted from Poppinga et al.^23^). The diamond chip with the sensitive diamond layer (1–2 μm thick)^22^, ^23^ defined by the Schottky contact is shown as a green line. The type of charge carriers, that is, positive or negative, collected depends on which detector's contact (blue or red) is connected to the electrometer.

### 2.B. OR measurements and small field output correction factors

The OR measurements were performed at a Varian TrueBeam linear accelerator (Palo Alto, USA) using a 6‐MV photon beam with 600 MU/min at a source‐to‐surface distance (SSD) of 90 cm in a MP3 water phantom (PTW Freiburg, Germany). The mD detectors were connected to the UNIDOSwebline electrometer (PTW Freiburg, Germany) and positioned axially at 10 cm depth. Measurements were performed using four mD detectors: one standard (mD_standard) and three modified detectors, as shown schematically in the right insert of Fig. 1. In the first modified mD detector (mD_reversed), the type of charge carriers collected is reversed by connecting the opposite contact to the electrometer. In the second modified mD detector (mD_shortened), the contacts have been shortened. The third modified mD detector (mD_noChip) is the same as the standard version but the diamond chip with the sensitive volume has been removed.

The OR were obtained for nominal field sizes from 6 mm × 6 mm to 40 mm × 40 mm. A plastic scintillation detector (Exradin W1, Standard Imaging, Middleton, USA) was used as reference detector to obtain the small field output factors, $D_{s_{\mathrm{eff}}}^{W1}/D_{40\mathrm{mm}}^{W1}$. The calibration of the scintillation detector to account for Cerenkov signal was performed according to a previous publication.^2^ The Cerenkov light ratio (CLR) was determined to be 0.655. Measurements were performed with the cylindrical axis of the detector aligned to the beam's axis. All measured OR were normalized to the field size of 40 mm × 40 mm. The measured small field output correction factors, *k*_meas_, were calculated using Eq. (5)

(5) $$k_{\mathrm{meas}}\left(s_{\mathrm{eff}}\right)=\frac{D_{s_{\mathrm{eff}}}^{W1}/D_{40\mathrm{mm}}^{W1}}{\left|Q_{s_{\mathrm{eff}}}^{+/-}\right|/\left|Q_{40\mathrm{mm}}^{+/-}\right|}$$

where $|Q_{s_{\mathrm{eff}}}^{+/-}|$ are the absolute signals obtained using the mD detectors. *s*_eff_ is effective dosimetric field side length computed as $\sqrt{x_{1/2}\cdot y_{1/2}}$, where *x*_1/2_ and *y*_1/2_ are the full width at half maximum values of the dose profiles in the *x* and *y* directions, respectively, at the measurement depth.

### 2.C. Monte Carlo simulations of OR and small field output correction factors

Simulations were performed using the EGSnrc package and the egs_chamber user‐code. The detector was modeled according to the manufacturer's blueprint including the metal contacts and the cable. The OR obtained using the mD detector was simulated using the IAEA phase space files of Varian Clinac iX 6 MV photon beam for field sizes of 5 mm × 5 mm, 10 mm × 10 mm, 20 mm × 20 mm, and 40 mm × 40 mm. The SSD was 100 cm and the detector, that is, its active layer was placed at 10 cm depth. The ECUT and PCUT values were chosen as 521 keV and 10 keV, respectively. The signal of the mD detector was recorded as |*Q* ^EDEP^|, which was computed by first scoring the EDEP within the sensitive volume of the mD detector (disk of 2.2 mm diameter and 1 μm thickness) and then converted to charge by dividing the energy deposited with the *w*‐value of diamond (13.2 eV) and multiplied by the elementary charge.

The simulated OR was normalized to the nominal field size of 40 mm × 40 mm. The field output factors, $D_{s_{\mathrm{eff}}}^{1\mathrm{mm}}/D_{40\mathrm{mm}}^{1\mathrm{mm}}$, were calculated using a 0.3 mm thick water cylinder of 1 mm diameter. The simulated small field output correction factors, *k*_sim_, were computed according to Eq. (6)

(6) $$k_{\mathrm{sim}}\left(s_{\mathrm{eff}}\right)=\frac{D_{s_{\mathrm{eff}}}^{1\mathrm{mm}}/D_{40\mathrm{mm}}^{1\mathrm{mm}}}{|Q_{s_{\mathrm{eff}}}^{\mathrm{EDEP}}\left|/\right|Q_{40\mathrm{mm}}^{\mathrm{EDEP}}|}$$

To compute the volume‐averaging correction factors, *P*_vol_, for the mD detector, the simulations of the field output factors were repeated using a 0.3 mm thick water cylinder with the same diameter as the sensitive volume of the detector, that is, 2.2 mm. The values of *P*_vol_ were computed according to Eq. (7)

(7) $$P_{\mathrm{vol}}\left(s_{\mathrm{eff}}\right)=\frac{D_{s_{\mathrm{eff}}}^{1\mathrm{mm}}/D_{40\mathrm{mm}}^{1\mathrm{mm}}}{D_{s_{\mathrm{eff}}}^{2.2\mathrm{mm}}/D_{40\mathrm{mm}}^{2.2\mathrm{mm}}}$$

### 2.D. Monte Carlo simulations of radiation‐induced charge imbalance

An adapted user‐code described in a previous work^27^ was used to calculate the quantities *Q* ^in^ and *Q* ^out^ in the metal contacts and cable of the mD detector according to Eq. (1). The Monte Carlo user‐code tracks each electron within the simulation geometry, where its new location after each transport step is registered. Each of these electron tracks is stored and later analyzed in terms of its start‐ and end‐point. The start point is the point of interaction, at which it is released, and the end‐point is either the point within the simulation geometry, at which the electron is stopped when its kinetic energy falls below the chosen cutoff of 1 keV, or at which it exits the simulation geometry. If the electron starts within and stopped outside the metal contacts or cable, the quantity *Q* ^out^ is increased by one elementary charge, that is, 1.6 × 10^−19^ C. If the secondary electron started outside and stopped within the metal contacts or cable, the quantity *Q* ^in^ is increased by one electron elementary charge (−1.6 × 10^−19^ C). The radiation‐induced net charge is then calculated according to Eq. (1). The quantity |*Q* ^EDEP^| was computed as described in Section 2.C. More details of the user‐code can be read in the previous work.^27^

Simulations were performed for the same setup as in the OR simulations using the same IAEA phase space files for the field sizes of 5 mm × 5 mm, 10 mm × 10 mm, 20 mm × 20 mm, and 40 mm × 40 mm.

## 3 Results

### 3.A. OR measurements with standard and modified mD detectors

Table 1 shows the raw measurement data of the W1 scintillation detector, the standard (mD_standard) and three modified versions of mD detector. Both the mD_standard and mD_shortened detectors have the same dose–response (1.0 nC/Gy), while the mD_reversed detector has a higher dose–response (1.3 nC/Gy). This variation in detector's sensitivity is mainly caused by the different thickness of the sensitive volume as shown by Marinelli et al.^22^ To ease comparison, the measured signals of the mD_reversed detector have been normalized to the dose–response of 1.0 nC/Gy (mD_reversed*). The corrected signals, that is, |*Q* ^EDEP^|, were computed according to Eqs. (2), (3), and (4).

**Table 1:** Raw measured signals, $Q_{s_{\mathrm{eff}}}^{+/-}$ in nC, of the mD detectors and W1 scintillator integrated over 60 s. The dose–response (nC/Gy) of each mD detector, obtained using a 10 cm × 10 cm 6 MV photon beam, is provided. |*Q* ^EDEP^| have been computed from Eqs. (2) and (3) by considering the signals of mD_noChip as *Q* ^net^ in Eq. (1); and from Eq. (4) by taking the mean of the absolute signals of the standard and mD_reversed* detectors

*s*_eff_/mm	6.4	8.2	10.2	16	20	30	40
W1	0.066	0.076	0.083	0.093	0.096	0.101	0.105
mD_standard (1.0 nC/Gy)	2.672	3.084	3.330	3.668	3.770	3.941	4.093
mD_reversed (1.3 nC/Gy)	−3.340	−3.904	−4.245	−4.718	−4.865	−5.095	−5.288
mD_reversed* (1.0 nC/Gy)	−2.569	−3.003	−3.265	−3.629	−3.742	−3.919	−4.068
mD_shortened (1.0 nC/Gy)	2.674	3.094	3.351	3.706	3.815	3.988	4.136
mD_noChip	0.0451	0.0335	0.0237	0.0106	0.0080	0.0073	0.0084
|*Q* ^EDEP^|							
Eq. (2): |mD_standard| − mD_noChip	2.627	3.050	3.306	3.657	3.762	3.934	4.085
Eq. (3): |mD_reversed*| + mD_noChip	2.614	3.037	3.289	3.640	3.750	3.926	4.076
Eq. (4): (|mD_standard| + |mD_reversed*|)/2	2.621	3.043	3.298	3.649	3.756	3.930	4.080

**Figure 2:**
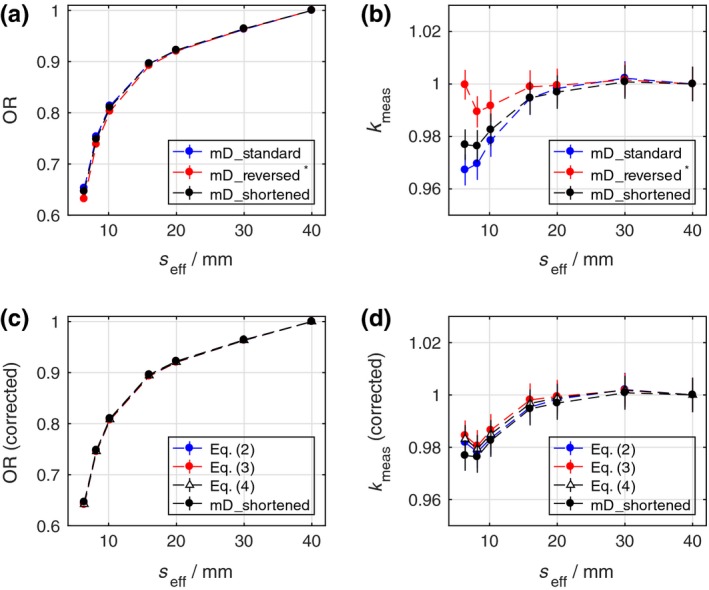
(a) Measured output ratio (OR) using the standard microDiamond (mD_standard) and two modified mD detectors (mD_reversed* and mD_shortened); (b) measured small field output correction factor, *k*_meas_, obtained using the OR in (a); (c) Corrected OR according to Eqs. (2), (3), and (4) and the OR measured using the modified detector with shortened contacts (mD_shortened); (d) small field output correction factor, *k*_meas_, obtained using the corrected OR in (c) and the OR measured with the mD_shortened detector. [Color figure can be viewed at http://www.wileyonlinelibrary.com]

The measured OR using the standard (mD_standard) and two modified mD detectors (mD_reversed* and mD_shortened) are shown in Fig. 2(a). At small field sizes, the OR measured with the mD_standard detector are higher than those measured with the modified mD detector with reversed contact (mD_reversed*). The corrected OR calculated from |*Q* ^EDEP^| according to Eqs. (2), (3), and (4) are shown in Fig. 2(c). All the corrected OR agree within 0.6% at the investigated field sizes. Good agreement was also found between the corrected OR and the OR measured using the modified mD detector with shortened contacts (mD_shortened).

The measured output correction factors, *k*_meas_, derived according to Eq. (5) from the OR in Fig. 2(a) are presented in Fig. 2(b). The correction of the mD_standard detector amounts to around 3.5% at the smallest field size investigated. This observed over‐response would correspond to the results reported in the literature so far for the commercial available version of the detector. On the other hand, the corrections of the mD_reversed* detector are within 1% at all investigated field sizes. This is a consequence of Eq. (3), where the charge imbalance will reduce the measured absolute signal partly compensating the density perturbation.

The measured output correction factors, *k*_meas_, derived from the corrected OR in Fig. 2(c) show an over‐response of <2% at the investigated field sizes. It is noteworthy that a turnaround point can now be observed between the field sizes of *s*_eff_ = 6.4 mm and 10.2 mm. By shortening its contacts, the mD_shortened detector also exhibits a smaller over‐response than the mD_standard detector with correction factors comparable to those computed from the corrected OR. Nevertheless, the small residual difference indicates that, although the effect of charge imbalance has been largely reduced, the signal of mD_shortened detector is still slightly influenced by the effect in the remaining parts of the contacts and cable.

### 3.B. Monte Carlo simulations of small field output correction factors

**Figure 3:**
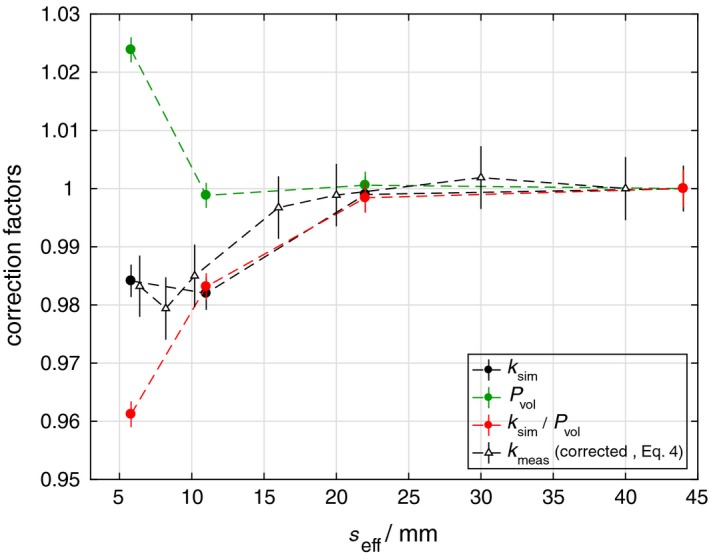
Simulated output correction factors, *k*_sim_, and the volume‐averaging correction factors, *P*_vol_, for the microDiamond (mD) detector obtained using the IAEA phase space files of Varian Clinac iX 6 MV photon beam. The ratios *k*_sim_/*P*_vol_ represent the correction for the remaining perturbation effects except for the volume‐averaging effect. The measured output correction factors, *k*_meas_, computed from the corrected output ratio (OR) according to Eq. (4) show good agreement with the simulated values. [Color figure can be viewed at http://www.wileyonlinelibrary.com]

The simulated output correction factors, *k*_sim_, computed according to Eq. (6) are presented in Fig. 3. The corrections due to the mD detector's over‐response are within 2% for the field sizes simulated. A similar trend as the results in Fig. 2(d) can be observed here, where the correction factors show a decreasing trend with decreasing field size up to a maximum over‐response of around 2% at *s*_eff_ = 11 mm with a turnaround point between *s*_eff_ = 5.5 mm and 11 mm. The upward trend of the correction factors at the smallest field size is mainly caused by a steep increase in the volume‐averaging correction factor, *P*_vol_, shown as green circles in Fig. 3. The remaining corrections due to other perturbation effects, except the volume‐averaging effect, were computed as the ratios *k*_sim_/*P*_vol_. As mentioned earlier, the density perturbation effect due to the structural components of the mD detector with density higher than normal water leads to an increasing over‐response at small field sizes. Nevertheless, this density perturbation effect is largely compensated by the volume‐averaging effect, reducing the over‐response of the mD detector at these small field sizes.

### 3.C. Quantitation of the effect of radiation induced charge imbalance

The measured absolute signal of a mD detector, |*Q* ^+^| or |*Q* ^−^|, can be described using Eqs. (2) or (3) in terms of the charge released by EDEP in the sensitive volume, |*Q* ^EDEP^|, and the charge imbalance in the contacts and cable, *Q* ^net^, neglecting other influence factors such as ion recombination and temperature effects. Consequently, the field size‐dependent ratios $|Q_{s_{\mathrm{eff}}}^{\mathrm{EDEP}}\left|/\right|Q_{s_{\mathrm{eff}}}^{+}|$ can be derived to quantify the effect of radiation‐induced charge imbalance. In fact, these ratios represent the corrections required to eliminate the effect of radiation‐induced charge imbalance from the measured signals of a standard mD detector, $|Q_{s_{\mathrm{eff}}}^{+}|$, to obtain $|Q_{s_{\mathrm{eff}}}^{\mathrm{EDEP}}|$. These ratios have been derived using three approaches in this work.

Firstly, $|Q_{s_{\mathrm{eff}}}^{\mathrm{EDEP}}|$ were obtained from Eq. (2) by considering the signal measured with the detector without the diamond chip (mD_noChip) as $Q_{s_{\mathrm{eff}}}^{\mathrm{net}}$. Therefore, the ratios $|Q_{s_{\mathrm{eff}}}^{\mathrm{EDEP}}\left|/\right|Q_{s_{\mathrm{eff}}}^{+}|$ can be calculated from the measured signal of the mD_standard and mD_noChip detectors according to Eq. (8).

(8) $$\frac{\left|Q_{s_{\mathrm{eff}}}^{\mathrm{EDEP}}\right|}{\left|Q_{s_{\mathrm{eff}}}^{+}\right|}=\frac{\left|Q_{s_{\mathrm{eff}}}^{+}\right|-Q_{s_{\mathrm{eff}}}^{\mathrm{net}}}{\left|Q_{s_{\mathrm{eff}}}^{+}\right|}$$

Secondly, the values $|Q_{s_{\mathrm{eff}}}^{\mathrm{EDEP}}|$ were derived according to Eq. (4) using the measured absolute signals of the mD_standard and mD_reversed* detectors. The ratios $|Q_{s_{\mathrm{eff}}}^{\mathrm{EDEP}}\left|/\right|Q_{s_{\mathrm{eff}}}^{+}|$ are then given by

(9) $$\frac{\left|Q_{s_{\mathrm{eff}}}^{\mathrm{EDEP}}\right|}{\left|Q_{s_{\mathrm{eff}}}^{+}\right|}=\frac{\left|Q_{s_{\mathrm{eff}}}^{+}\right|+\left|Q_{s_{\mathrm{eff}}}^{-}\right|}{2\left|Q_{s_{\mathrm{eff}}}^{+}\right|}$$

Thirdly, the quantities $|Q_{s_{\mathrm{eff}}}^{\mathrm{EDEP}}|$ and $Q_{s_{\mathrm{eff}}}^{\mathrm{net}}$ obtained directly from the Monte Carlo simulations described in Section 2.D were used to compute the ratio $|Q_{s_{\mathrm{eff}}}^{\mathrm{EDEP}}\left|/\right|Q_{s_{\mathrm{eff}}}^{+}|$, where $|Q_{s_{\mathrm{eff}}}^{+}\left|=\right|Q_{s_{\mathrm{eff}}}^{\mathrm{EDEP}}|+Q_{s_{\mathrm{eff}}}^{\mathrm{net}}$.

**Figure 4:**
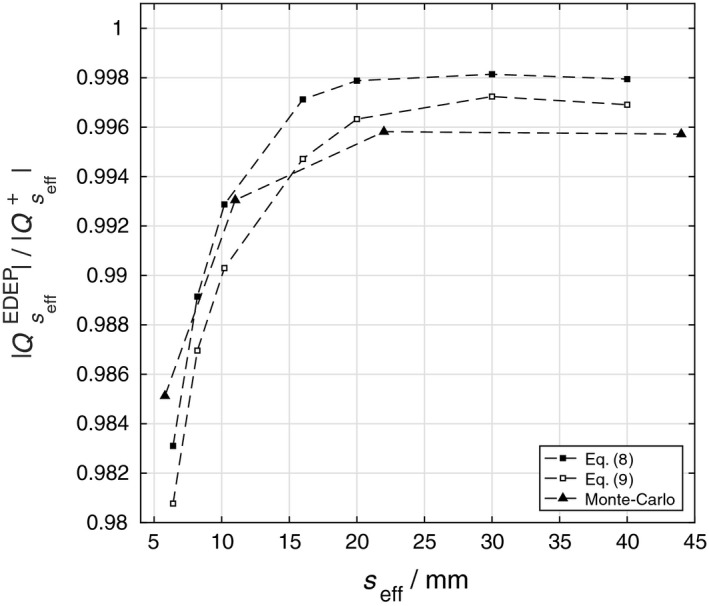
The field size dependent ratio $|Q_{s_{\mathrm{eff}}}^{\mathrm{EDEP}}\left|/\right|Q_{s_{\mathrm{eff}}}^{+}|$ computed from measurements according to Eqs. (8), (9) and using Monte Carlo simulations.

The results from the three approaches have been presented in Fig. 4. At the smallest field size investigated, the correction of the radiation‐induced charge imbalance amounts up to 2%. The results from the Monte Carlo simulations confirm that the ratios $|Q_{s_{\mathrm{eff}}}^{\mathrm{EDEP}}\left|/\right|Q_{s_{\mathrm{eff}}}^{+}|$ are field size dependent and increase with decreasing field size.

It is noteworthy that the values presented in Fig. 4 apply to mD detectors with a dose–response of 1 nC/Gy (10 cm × 10 cm 6 MV photon beam). Since the net radiation‐induced charge imbalance can be assumed to be the same for all mD detectors as the components in which the charge imbalance occurs are identical, the influence of radiation imbalance toward the detector's signal will depend on its sensitivity. In other words, the mD detector with a higher sensitivity will be less subjected to the effect of radiation‐induced charge imbalance.

## 4 Discussion

### 4.A. The field size dependence of charge imbalance in detector's components

Figures 2 and 4 show that the influence of the radiation‐induced charge imbalance in the metal contacts and cable of the mD detector exhibits a significant field size dependence at *s*_eff_ < 20 mm. Similar behavior has been reported for the polarity effect of microchambers, where the associated polarity effect is shown to increase with decreasing field size when the chambers are oriented axially, that is, with their axes parallel to the beam's axis.^27^ In fact, the underlying mechanisms for these observations of the mD detector and ionization chambers are the same.

As shown in Fig. 4, the effect remains almost constant at large field sizes (*s*_eff_ > 20 mm), which indicates that the radiation‐induced charge within the contacts and cable is, up to a small residual difference, balanced resulting in minimal radiation induced net charge according to Eq. (1). On the other hand, at small field sizes, since less electrons will be released in the surrounding medium that are then stopped in the contacts and cable, the compensation effect observed for large field sizes is deficient giving rise to the field size dependence of the effect. In fact, for the field sizes investigated, the irradiation of these components always causes excess of holes within the contacts and cable when the detector is positioned along the central axis, that is, the value of *Q* ^net^ is always positive. Although both the contacts depicted in Fig. 1 (red and blue) are equally subjected to the same effect, only the charge imbalance in the contact that is connected to the electrometer will affect the measured signal. Furthermore, due to the decreasing ionization signal in the sensitive detector volume caused by the reduced machine output at small field sizes, the impact of radiation‐induced net charge will also become more dominant.

### 4.B. Small field perturbation effects

Monte Carlo simulations have demonstrated that the dose–response of the mD detector in small fields is subjected to the volume‐averaging effect and the perturbation of the electron fluence in the detector's sensitive volume, which can be corrected altogether using the small field output correction factors. Figure 3 shows that the corrections for the volume‐averaging effect increase steeply at *s*_eff_ smaller than 10 mm causing the mD detector to under‐respond. On the other hand, the perturbation of the electron fluence that is mainly caused by the detector's structural components with electron density higher than normal water, as discussed by several authors,^12^, ^13^, ^15^ causes the mD detector to over‐respond. Although the atomic composition of these components will also result in a perturbation of the electron fluence,^29^ this effect was shown to be constant over the field size and hence not affecting the small field output correction factors significantly.^15^ As demonstrated in Fig. 3, the density perturbation effect overweighs the volume‐averaging effect at the small field sizes investigated resulting in output correction factors down to 0.98.

However, experimental data have suggested more prominent over‐response behavior of the mD detector. The results from this study have demonstrated that the discrepancy between experimental and Monte Carlo output correction factors can be attributed to the radiation‐induced charge imbalance in the metal contacts and cable of the mD detector. This effect will cause a standard mD detector to over‐respond by an additional of 1.5 to 2% during OR measurements at the smallest field size studied here. Therefore, output correction factors derived directly from these experimental OR so far would have incorporated the effect of charge imbalance. In other words, if experimental output correction factors are used to correct for OR measurements obtained with the mD detector, no further correction for charge imbalance is required. These values are presented, for example, in Fig. 2(b) obtained with the standard mD detector, which agree with the values recommend in the TRS 483^19^ within 0.8%. However, if Monte Carlo simulated output correction factors are preferred, such as those presented in Fig. 3 (black circles), the measured signal of the mD detector must be corrected for the effect of charge imbalance separately, for example, by using the values presented in Fig. 4, before the Monte Carlo output correction factors can be applied.

### 4.C. Strategy to decrease the effect of charge imbalance

Detailed analysis of the results from the Monte Carlo simulations described in Section 3.C have provided additional information on the contribution of each detector component toward the resulting field size‐dependent charge imbalance. Hence, the components with significant contributions can be identified and modified with the aim to reduce this effect. Based on these insights, a mD detector has been modified, where the contacts have been shortened (mD_shortened in this work). As demonstrated in Fig. 2(c), the measured OR with this modified mD detector show good agreement with the corrected OR, indicating that the effect of charge imbalance has been largely removed. This modified mD detector will therefore require less correction in small field dosimetry.

## 5 Conclusions

The role of radiation‐induced charge imbalance in the detector's components on the dose–response of the mD detector at small field sizes has been quantified using standard and modified versions of the detectors, as well as using Monte Carlo simulations. The discrepancy between experimental and Monte Carlo simulated output correction factors observed so far diminishes after the effect of charge imbalance has been accounted for in the experimental results. Component analysis using detailed Monte Carlo simulations has been performed to identify the structural components contributing significantly to this effect. A modified mD detector constructed based on these insights has shown favorable behavior requiring only corrections of <2% at the small field sizes investigated. The findings in this work also demonstrate the importance to account for charge imbalance in the Monte Carlo simulation of semiconductor detectors.

## Conflicts of interest

Daniela Poppinga, Jan Würfel, Dennis Vogt, and Rafael Kranzer are employees of PTW Freiburg. The other authors have no conflicts of interest to report.

## References

[1] Azangwe G , Grochowska P , Georg D , et al. Detector to detector corrections: a comprehensive experimental study of detector specific correction factors for beam output measurements for small radiotherapy beams. Med Phys. 2014;41:072103. 24989398 10.1118/1.4883795

[2] Poppinga D , Delfs B , Meyners J , Harder D , Poppe B , Looe HK . The output factor correction as function of the photon beam field size – direct measurement and calculation from the lateral dose response functions of gas‐filled and solid detectors. Z Med Phys. 2017 28:224–235. 28869164 10.1016/j.zemedi.2017.07.006

[3] Ralston A , Tyler M , Liu P , McKenzie D , Suchowerska N . Over‐response of synthetic microDiamond detectors in small radiation fields. Phys Med Biol. 2014;59:5873–5881. 25211368 10.1088/0031-9155/59/19/5873

[4] Chalkley A , Heyes G . Evaluation of a synthetic single‐crystal diamond detector for relative dosimetry measurements on a CyberKnife. Br J Radiol. 2014;87:20130768. 24588671 10.1259/bjr.20130768 PMC4064610

[5] Andreo P , Palmans H , Marteinsdottir M , Benmakhlouf H , Carlsson‐Tedgren A . On the Monte Carlo simulation of small‐field micro‐diamond detectors for megavoltage photon dosimetry. Phys Med Biol. 2015;61:L1–L10. 26630437 10.1088/0031-9155/61/1/L1

[6] Francescon P , Kilby W , Noll JM , Masi L , Satariano N , Russo S . Monte Carlo simulated corrections for beam commissioning measurements with circular and MLC shaped fields on the CyberKnife M6 System: a study including diode, microchamber, point scintillator, and synthetic microdiamond detectors. Phys Med Biol. 2017;62:1076–1095. 28033110 10.1088/1361-6560/aa5610

[7] Papaconstadopoulos P , Tessier F , Seuntjens J . On the correction, perturbation and modification of small field detectors in relative dosimetry. Phys Med Biol. 2014;59:5937–5952. 25210930 10.1088/0031-9155/59/19/5937

[8] Morales JE , Crowe SB , Hill R , Freeman N , Trapp JV . Dosimetry of cone‐defined stereotactic radiosurgery fields with a commercial synthetic diamond detector. Med Phys. 2014;41:111702. 25370616 10.1118/1.4895827

[9] Larraga‐Gutierrez JM , Ballesteros‐Zebadua P , Rodriguez‐Ponce M , Garcia‐Garduno OA , de la Cruz OO . Properties of a commercial PTW‐60019 synthetic diamond detector for the dosimetry of small radiotherapy beams. Phys Med Biol. 2015;60:905–924. 25564826 10.1088/0031-9155/60/2/905

[10] O'Brien DJ , Leon‐Vintro L , McClean B . Small field detector correction factors kQclin, Qmsr (fclin, fmsr) for silicon‐diode and diamond detectors with circular 6 MV fields derived using both empirical and numerical methods. Med Phys. 2016;43:411. 26745934 10.1118/1.4938584

[11] Underwood TS , Rowland BC , Ferrand R , Vieillevigne L . Application of the Exradin W1 scintillator to determine Ediode 60017 and microDiamond 60019 correction factors for relative dosimetry within small MV and FFF fields. Phys Med Biol. 2015;60:6669–6683. 26271097 10.1088/0031-9155/60/17/6669

[12] Looe HK , Delfs B , Poppinga D , Jiang P , Harder D , Poppe B . The ‘cutting away’ of potential secondary electron tracks explains the effects of beam size and detector wall density in small‐field photon dosimetry. Phys Med Biol. 2017;63:015001. 29148434 10.1088/1361-6560/aa9b46

[13] Looe HK , Harder D , Poppe B . Understanding the lateral dose response functions of high‐resolution photon detectors by reverse Monte Carlo and deconvolution analysis. Phys Med Biol. 2015;60:6585–6607. 26267311 10.1088/0031-9155/60/16/6585

[14] Looe HK , Harder D , Poppe B . The energy dependence of the lateral dose response functions of detectors with various densities in photon‐beam dosimetry. Phys Med Biol. 2016;62:N32–N44. 27992384 10.1088/1361-6560/aa54aa

[15] Fenwick JD , Georgiou G , Rowbottom CG , Underwood TSA , Kumar S , Nahum AE . Origins of the changing detector response in small megavoltage photon radiation fields. Phys Med Biol. 2018;63:125003. 29757158 10.1088/1361-6560/aac478

[16] Fenwick JD , Kumar S , Scott AJ , Nahum AE . Using cavity theory to describe the dependence on detector density of dosimeter response in non‐equilibrium small fields. Phys Med Biol. 2013;58:2901–2923. 23574749 10.1088/0031-9155/58/9/2901

[17] Scott AJ , Kumar S , Nahum AE , Fenwick JD . Characterizing the influence of detector density on dosimeter response in non‐equilibrium small photon fields. Phys Med Biol. 2012;57:4461–4476. 22722374 10.1088/0031-9155/57/14/4461

[18] De Coste V , Francescon P , Marinelli M , et al. Is the PTW 60019 microDiamond a suitable candidate for small field reference dosimetry? Phys Med Biol. 2017;62:7036–7055. 28791962 10.1088/1361-6560/aa7e59

[19] IAEA Technical Reports Series No. 483 . Dosimetry of Small Static Fields Used in External Beam Radiotherapy An International Code of Practice for Reference and Relative Dose Determination. 10.1002/mp.13208 30247757

[20] Crop F , Reynaert N , Pittomvils G , et al. The influence of small field sizes, penumbra, spot size and measurement depth on perturbation factors for microionization chambers. Phys Med Biol. 2009;54:2951–2969. 19384005 10.1088/0031-9155/54/9/024

[21] Bouchard H , Seuntjens J , Duane S , Kamio Y , Palmans H . Detector dose response in megavoltage small photon beams. I. Theoretical concepts. Med Phys. 2015;42:6033. 26429279 10.1118/1.4930053

[22] Marinelli M , Prestopino G , Verona C , Verona‐Rinati G . Experimental determination of the PTW 60019 microDiamond dosimeter active area and volume. Med Phys. 2016;43:5205. 27587052 10.1118/1.4961402

[23] Poppinga D , Delfs B , Meyners J , et al. Determination of the active volumes of solid‐state photon‐beam dosimetry detectors using the PTB proton microbeam. Med Phys. 2018;45:3340–3348. 29727482 10.1002/mp.12948

[24] Butler DJ , Beveridge T , Lehmann J , Oliver CP , Stevenson AW , Livingstone J . Spatial response of synthetic microDiamond and diode detectors measured with kilovoltage synchrotron radiation. Med Phys. 2018;45:943–952. 29244899 10.1002/mp.12733

[25] Das IJ , Francescon P . Comments on the TRS‐483 protocol on small field dosimetry. Med Phys. 2018;45:5666–5668. 30536942 10.1002/mp.13236

[26] Palmans H , Andreo P , Huq MS , Seuntjens J , Christaki KE , Meghzifene A . Reply to “comments on the TRS‐483 *protocol on small field* dosimetry” [Med. Phys. 45(12), 5666‐5668 (2018)]. Med Phys. 2018;45:5669–5671. 30536943 10.1002/mp.13235

[27] Looe HK , Büsing I , Tekin T , et al. The polarity effect of compact ionization chambers used for small field dosimetry. Med Phys. 2018;45:5608–5621. 30294821 10.1002/mp.13227

[28] Canali C , Gatti E , Kozlov SF , et al. Electrical properties and performances of natural diamond nuclear radiation detectors. Nucl Instrum Methods. 1979;160:73–77.

[29] Andreo P , Benmakhlouf H . Role of the density, density effect and mean excitation energy in solid‐state detectors for small photon fields. Phys Med Biol. 2017;62:1518–1532. 28036305 10.1088/1361-6560/aa562e

